# FET-PET radiomics in recurrent glioblastoma: prognostic value for outcome after re-irradiation?

**DOI:** 10.1186/s13014-020-01744-8

**Published:** 2021-03-03

**Authors:** Montserrat Carles, Ilinca Popp, Michael Maximilian Starke, Michael Mix, Horst Urbach, Tanja Schimek-Jasch, Franziska Eckert, Maximilian Niyazi, Dimos Baltas, Anca L. Grosu

**Affiliations:** 1 Division of Medical Physics, Department of Radiation Oncology, Medical Center, University of Freiburg, Robert-Koch Str. 3, 79106 Freiburg, Germany; 2 German Cancer Consortium (DKTK), German Cancer Research Center (DKFZ), Partner Site Freiburg, Heidelberg, Germany; 3 Biomedical Imaging Research Group (GIBI230-PREBI), Imaging La Fe Node at Distributed Network for Biomedical Imaging (ReDIB) Unique Scientific and Technical Infrastructures (ICTS), La Fe Health Research Institute, Valencia, Spain; 4 Department of Radiation Oncology, Medical Center, Faculty of Medicine, University of Freiburg, Freiburg, Germany; 5 Department of Nuclear Medicine, Medical Center, Faculty of Medicine, University of Freiburg, Freiburg, Germany; 6 Department of Neuroradiology, Medical Center, Faculty of Medicine, University of Freiburg, Freiburg, Germany; 7 Department of Radiation Oncology, University Hospital Tübingen, Tübingen, Germany; 8 German Cancer Consortium (DKTK), German Cancer Research Center (DKFZ), Partner Site Tübingen, Tübingen, Germany; 9 Department of Radiation Oncology, University Hospital, LMU Munich, Munich, Germany; 10 Cerman Cancer Consortium (DKTK), German Cancer Research Center (DKFZ), Partner Site Munich, Munich, Germany

**Keywords:** Recurrent-glioblastoma, FET-PET, Radiomics, Re-irradiation

## Abstract

**Purpose:**

The value of O-(2-[18F]fluoroethyl)-L-tyrosine (FET)-positron emission tomography (PET)-radiomics in the outcome assessment of patients with recurrent glioblastoma (rGBM) has not been evaluated until now. The aim of this study was to evaluate whether a prognostic model based on FET-PET radiomics features (RF) is feasible and can identify rGBM patients that would most benefit from re-irradiation.

**Methods:**

We prospectively recruited rGBM patients who underwent FET-PET before re-irradiation (GLIAA-Pilot trial, DRKS00000633). Tumor volume was delineated using a semi-automatic method with a threshold of 1.8 times the standardized-uptake-value of the background. 135 FET-RF (histogram parameters, shape and texture features) were extracted. The analysis involved the characterization of tumor and non-tumor tissue with FET-RF and the evaluation of the prognostic value of FET-RF for time-to-progression (TTP), overall survival (OS) and recurrence location (RL).

**Results:**

Thirty-two rGBM patients constituted our cohort. FET-RF discriminated significantly between tumor and non-tumor. The texture feature Small-Zone-Low-Gray-Level-Emphasis (SZLGE) showed the best performance for the prediction of TTP (*p* = 0.001, satisfying Bonferroni-multiple-test significance level). Additionally, two radiomics signatures could predict TTP (TTP-radiomics-signature, *p* = 0.001) and OS (OS-radiomics-signature, *p* = 0.038). SZLGE and the TTP-radiomics-signature additionally predicted RL. Specifically, high values for TTP-radiomics-signature and for SZLGE indicated not only earlier progression, but also a RL within the initial FET-PET active volume.

**Conclusion:**

Our findings suggest that FET-PET radiomics could contribute to the prognostic assessment and selection of rGBM-patients benefiting from re-irradiation.

*Trial registration* DRKS00000633. Registered on 8th of December in 2010.

https://www.drks.de/drks_web/navigate.do?navigationId=trial.HTML&TRIAL_ID=DRKS00000633.

## Introduction

Surgery, radiation therapy (RT) and chemotherapy is the standard treatment in glioblastoma (GBM) [1]. In recurrent glioblastoma (rGBM), re-irradiation (re-RT) is an important therapeutic alternative, which may delay further disease progression and improve survival [2, 3]. However, the re-RT can be associated with a high risk of brain edema, radiation necrosis, increased dose of corticosteroids and impaired quality of life. Therefore, the selection of patients who will most probably benefit from this treatment is extremely important. However, apart from general clinical criteria, such as primary histology, methylation, performance status, time between first and second RT and age [4, 5], there are currently only few studies showing that imaging biomarkers could predict treatment outcome after re-RT in rGBM [6–8].

Generally, diagnosis, treatment planning and follow-up of GBM are based on magnetic resonance imaging (MRI): gadolinium contrast enhanced T1-weighted images (Gd-T1MR), T2 images, FLAIR images etc. Contrast enhancement is facilitated by a disruption of the blood–brain-barrier, highly correlating with malignant tumor tissue. Nevertheless, contrast enhancement could also occur after a recent surgery, RT or chemotherapy (pseudoprogression). In this case, amino-acid positron emission tomography (PET) has been proven to be able to non-invasively differentiate treatment-related changes from real tumor progression [8–10]. Consequently, the use of the radiolabeled amino acid O-(2-[18F]fluoroethyl)-L-tyrosine (FET) has rapidly increased in the last decade [9–14].

Research focused on the application of radiomics at different cancer sites has seen a significant boost in the past years. The aim of radiomics is the development of models based on the analysis of quantitative features (first and higher order statistics) derived from different imaging modalities [15, 16]. For gliomas, most of the radiomics publications have focused on MRI [17, 18]. Although only few recent studies have involved FET-PET image analysis, they showed the added value of FET-PET in the classification of tumor grades [19] and in predicting IDH genotype [20].

To the best of our knowledge, the prognostic role of FET-PET radiomics in patients with rGBM scheduled for re-irradiation has not yet been evaluated. The aim of the current study was to assess the feasibility of developing a model based on FET radiomics features (RF), with the objective of selecting rGBM patients that would most benefit from re-irradiation.

## Materials and methods

### Patients

Our study involved patients with rGBM (WHO grade IV) who had undergone standard radiochemotherapy after primary diagnosis. Thirty-two patients were prospectively enrolled in the open label mono-center pilot trial (DRKS00000633) of the GLIAA trial (NOA 10/ARO 2013-1) [21]. The clinical information for the patient cohort is summarized in Table 1. For all patients, the time between the first RT and re-RT was at least 6 months and the recurrent tumor was visible on baseline FET-PET with a diameter ranging from 1 to 6 cm. High-precision re-irradiation was performed in a stereotactic setup according to clinic standards. Dose specifications, contouring and constraints for organs at risk were identical to the ongoing GLIAA trial [21]. The prescribed dose was 39 Gy, 3 Gy/d, 5x/week and was set to ensure coverage of at least 95% of the planning target volume. Progression was diagnosed on conventional MRI in interdisciplinary tumor conferences of the Comprehensive Cancer Center Freiburg, by taking the updated RANO-criteria [22] and the patients’ clinical condition into consideration. Additional FET-PET and/or histological confirmation were used in unclear progression cases. Time-to-tumor-progression (TTP) and overall survival (OS) were defined as the times between the start of the re-irradiation treatment and the first progression for TTP and death for OS. Recurrence location (RL) distinguished between inside (> 50% volume) or outside the initial FET-PET active volume (see V_PET_ definition in subsection “Segmentation”).

**Table 1:** Clinical information of the patient cohort. TTP: time interval between the start of the re-irradiation treatment and the first progression

	Patient cohort (n = 32)
Age (years, median, range)	52 (30–77)
Sex	
Male	17 (53.1%)
Female	15 (46.9%)
GBM	
Unifocal	18 (56.3%)
Multifocal	14 (43.7%)
IDH-mutation	
Mutated	10 (31.2%)
Wild-type	14 (43.8%)
Unknown	8 (25%)
MGMT-status of recurrent tumor	
Methylated Not methylated Unknown	7 (21.9%) 8 (25%) 17 (53.1%)
Surgery of recurrent tumor before re-irradiation	
Yes No	25 (78.1%) 7 (21.9%)
Time to progression (TTP) (days, median, range)	91 (18–405)
Overall survival (OS) (days, median, range)	296.5 (18–1334)

*OS* time interval between the start of re-irradiation and death

### FET-PET imaging

The ^18^F-labeled amino acid was synthesized via [^18^F]-fluoroalkylation of tyrosine with a specific activity larger than 18.5 GBq/μmol. The FET-PET acquisition protocol for our patient cohort was defined as follows: a static 15 min scan was performed 20 min post intravenous injection of 200–300 MBq FET. Scans were performed on two different PET/CT systems from Philips (Netherlands): GEMINI TF TOF 64 (TF64) and GEMINI TF 16 Big Bore (BB). BB was employed for 14 of the 32 patients. The scanners fulfilled the requirements indicated in the European Association of Nuclear Medicine (EANM) imaging guidelines and obtained EANM Research Ltd. (EARL) accreditation. The transverse spatial resolution at 1 cm from the central axis of the scanner was 4.8 mm. PET data was corrected for random coincidences, scatter and attenuation, based on the corresponding CT dataset. The reconstruction methods was a LOR-based ordered-subset iterative time-of-flight algorithm using spherical coordinates (BLOB) with three iterations, 33 subsets and a relaxation parameter for smoothing of 0.35. PET images had a voxel size of 2 × 2 × 2 mm^3^ and were normalized to decay-corrected injected activity per kg body weight (standardized-uptake-value SUV [g/ml]).

### Segmentation

**Fig.1:**
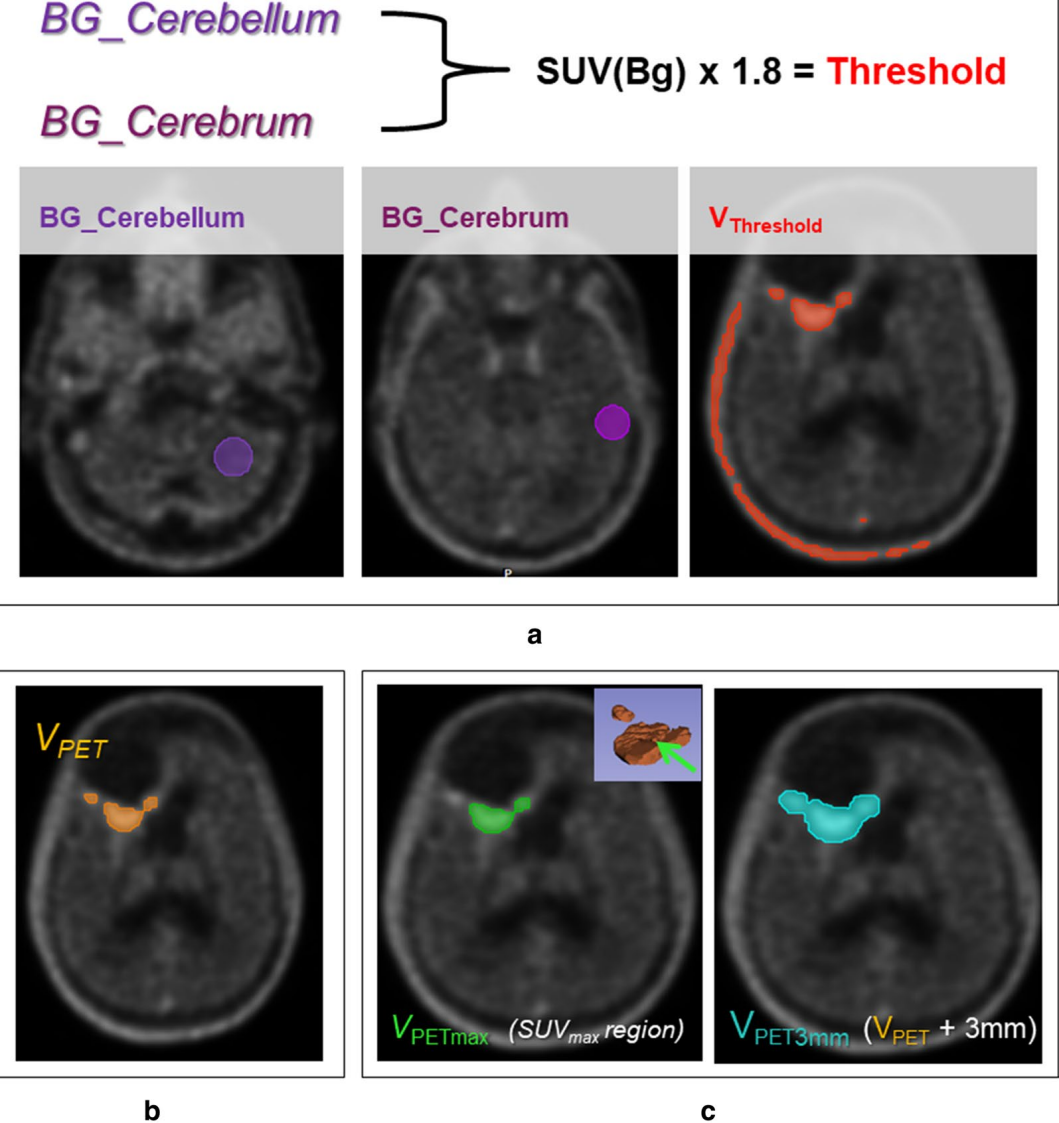
FET-PET segmentation steps: **a** for each patient a segmentation (V_Threshold_) was performed by a threshold of 1.8 times the FET uptake in background SUV(Bg.), with background defined by two spheres placed manually in cerebrum and cerebellum; **b** a radiation oncologist expert removed the regions which were not tumor-relevant and **c** two segmentations were defined on the remaining volume: V_PET3mm_ resulted from applying a margin of 3 mm around the whole PET volume (V_PET_) and V_PETmax_ was defined as the contiguous volume containing SUV_max_

Delineation on FET-PET took place in three steps (Fig. 1). Firstly, two spheres of constant diameter were manually placed in cerebrum and cerebellum. SUV of the background, SUV(Bg.), was defined by means of the average SUV values derived from the two spheres. Secondly, a threshold defined as 1.8 × SUV(Bg.) was applied. From the resulting volume (V_Threshold_), an experienced radiation oncologist removed the regions that were considered not tumor-related (e.g. blood vessels and extra cerebral enhancement), based on anatomical information conveyed by MRI and CT. Finally, from the remaining volume (V_PET_) two different contours (V_PETmax_ and V_PET3mm_) were generated. For the volume V_PET3mm_, a margin of 3 mm was applied to V_PET_. It was defined to cover all regions of FET uptake as well as the surrounding volume that could contain information concerning microscopic tumor cell spread, equivalent to the clinical target volume used for RT. For the high-risk volume defined as V_PETmax_, only the contiguous contour containing the maximum SUV was considered. The analysis of the V_PETmax_ was based on the hypothesis that the region with higher FET-uptake should be the most metabolically and mitotically active one and could therefore provide prognostic information concerning radiotherapy outcome.

### Radiomics features extraction

135 RF were computed using in-house software based on MATLAB® (The MathWorks Inc., Natick, MA). Definition of RF was done according to the 3D definition from Image Biomarker Standardization Initiative [23]. Histogram based statistics were validated with the Medical Imaging Interaction Toolkit (MITK) [24]. The validation of geometric and texture features was done with an open source code [25]. For texture feature computation, SUV values of the voxels within the contour were discretized with a fixed bin width (W = 0.01) [26, 27], resulting in different numbers of bins (from 35 to 936, with average 237) depending on the range of SUV values in the contour. Texture features were derived from five matrices: the 3D version of the gray-level co-occurrence matrix (GLCM [28, 29]); the gray-level run length matrix (GLRLM [30, 31]), the gray-level size zone matrix (GLSZM [30, 32]) and the neighborhood gray tone difference matrix (NGTDM [33]). In addition, on the voxel intensities within the contour we applied: (i) a Wavelet band-pass filtering (WF), with a weight ratio 1:2 between band-pass sub-bands and other sub-bands and (ii) an equal-probability quantization algorithm (Q), by using the function *histeq* of MATLAB®. The RF used in this study are listed in the Additional file 1: Table S1.

### Radiomics features selection: experimental phantom evaluation

In order to avoid misinterpretation of the results derived from our investigation, experimental phantom evaluation was performed to select the RF that should be included in the different parts of our analysis. In the analysis of the prognostic value of FET-RF an experimental phantom evaluation was necessary to identify the RF robust to the different PET/CT systems employed in our cohorts. In the analysis of RF for tumor characterization, we compared volumes with different sizes, i.e. RF computed in tumor against RF computed in a 4 cc-sphere within the background. It was therefore necessary to identify the RF independent of the size of the volume (number of voxels within the contour). For the experimental evaluation we employed the PET images resulted from the EARL (ResEARch for Life – European Association of Nuclear Medicine initiative) accreditation measurement of the NEMA NU2 Phantom (NP). To analyze the robustness of RF for different PET/CT systems 18 contours were delineated: 12 spherical contours (5.7–8.4 cc) were manually delineated in the background of NP and the 6 fillable spheres of the phantom (0.5–25 cc) were segmented by applying a threshold of 40% of the maximum uptake within the sphere. To analyze the intrinsic dependency of RF with the volume 102 contours (0.8–234 cc) were delineated in the background of the NP.

### Statistical analysis

The statistical analysis was performed using in-house software based on Wolfram Mathematica v 11.2. Wilcoxon signed rank test was used when comparing two data samples. Correlations were analyzed in terms of Spearman's correlation tests and strong correlation was identified by *p* < 0.05 and r > 0.8. For TTP and OS analysis, Kaplan–Meier curves were estimated and comparison between groups was evaluated with the log-rank test. Multivariate Cox regression was used for estimation of hazard ratios (HR) with 95% CI. For modelling with a binary output, an open source multivariate binary logistic regression analysis [25] was performed involving imbalance-adjusted bootstrap resampling (i.e. a resampling method alternative to the cross validation) in prediction performance estimation and computation of model coefficients. To correct for multiple test comparisons, the Bonferroni correction method was applied: the significance level was lowered to a value *p* < α/K, where K is the number of comparisons and α is the significance level set to 0.05.

## Results

### Radiomics features selection: experimental phantom evaluation

Results of the experimental phantom evaluation are summarized in the Additional file 2: Table S2. 61% RF were robust (Wilcoxon signed rank test) to the different PET/CT systems and 53% RF had no strong correlation (Spearman´s correlation test) with the number of voxels within the contour.

### FET radiomics features for tumor characterization

RF computed on V_PETmax_ and V_PET3mm_ were compared with respect to the RF computed on the 4 cc-sphere defined as background in cerebrum (V_Bg_). The impact of the intrinsic dependency of some RF on the size was minimized by: (i) using the same size for V_Bg_ in all patients and (ii) rejecting the RF with intrinsic dependence on size, based on results derived from an experimental phantom evaluation (see subsection *Radiomics features selection: experimental phantom evaluation*)*.* Consequently, only the RF that did not show statistically significant strong correlation with the size were involved in the current analysis. 75% of RF showed significant differences (Wilcoxon signed rank test, Bonferroni) between V_PETmax_ and V_Bg_. For V_PET3mm_, only 21% of RF had statistically different values with respect to V_Bg_. Detailed results are listed in Additional file 2: Table S2.

### ***Added value of radiomics features with respect to conventional indices (SUV***_***max***_*** and volume)***

For our patient cohort, we evaluated the added value of FET-RF with respect to the conventional indices SUV_max_ and volume. Overall, 64 RF for V_PETmax_ and 60 RF for V_PET3mm_ were simultaneously independent of volume and SUV_max_. Results are presented in Additional file 2: Table S2.

### Prognostic value of FET radiomics features

#### Univariate analysis

To avoid misinterpretation of the results, only RF that have been proven in the phantom evaluation to be robust to the different PET/CT scanners were included (see subsections *Radiomics features selection: experimental phantom evaluation*). In Table 2 the RF with the best Kaplan–Meier curve performance (log-rank test: *p* < 0.05) are presented. Overall, only the texture feature Small-Zone-Low-Gray-Emphasis (SZGLE) could predict earlier time-to-tumor progression (TTP) when the significance level was lowered by the Bonferroni multiple-test correction (statistically significant for *p* < 0.0013).

**Table 2:** Radiomics features derived from the two contours (V_PETmax_ and V_PET3mm_) showing significant *p* values < 0.05 in the Log-rank test for the Kaplan–Meier curves of overall-survival (OS) and time-to-tumor-progression (TTP)

		Correlation with overall survival (OS): *p* value		Correlation with time-to-progression (TTP): *p* value
Radiomics features extracted from V_PETmax_	SUV_min_	0.038	SZLGE	*0.001*
			Busyness	0.006
			WF_TS	0.012
			QVariance_CM_	0.029
Radiomics features extracted from V_PET3mm_	SUV_mean_	0.041	Eccentricity	0.004
	GLV	0.033		
	GLV2	0.011		
	WF_GLV	0.002		
	QAcor	0.013		
	QHGZE	0.013		
	QSZHGE	0.013		
	QGLN2	0.033		
	QHGRE	0.008		
	QSRHGE	0.008		
	QLRHGE	0.008		

Italics represent *p* values lower than the significance level lowered by Bonferroni multiple-test correction

#### Multivariate analysis: radiomics-signatures

To build radiomics-signatures, i.e. combinations of RF for the prognosis of OS and for TTP, we focused our analysis on the RF presented in Table 2, i.e. the RF robust to the PET/CT scanner and with the best Kaplan–Meier performance for the univariate analysis. To remove redundancy, we assessed the correlations between these RF. From each of the resulted correlated-feature groups, only the RF with more statistically significant prediction (lower *p* value in log-rank test), was selected. These RF were combined (radiomics-signature) into a multivariate Cox proportional hazards regression model for prediction of OS and of TTP. The resulting radiomics-signatures consisted:

(i) for the prediction of OS (OS-radiomics-signature) in the combination of SUV_mean_, WF_GLV and QLRHGE for V_PET3mm_ and SUV_min_ for V_PETmax_
(ii) for the prediction of TTP (TTP-radiomics-signature) in the combination of SZLGE, Busyness and QVariance_CM_ for V_PETmax_ and Eccentricity for V_PET3mm_.

**Fig. 2:**
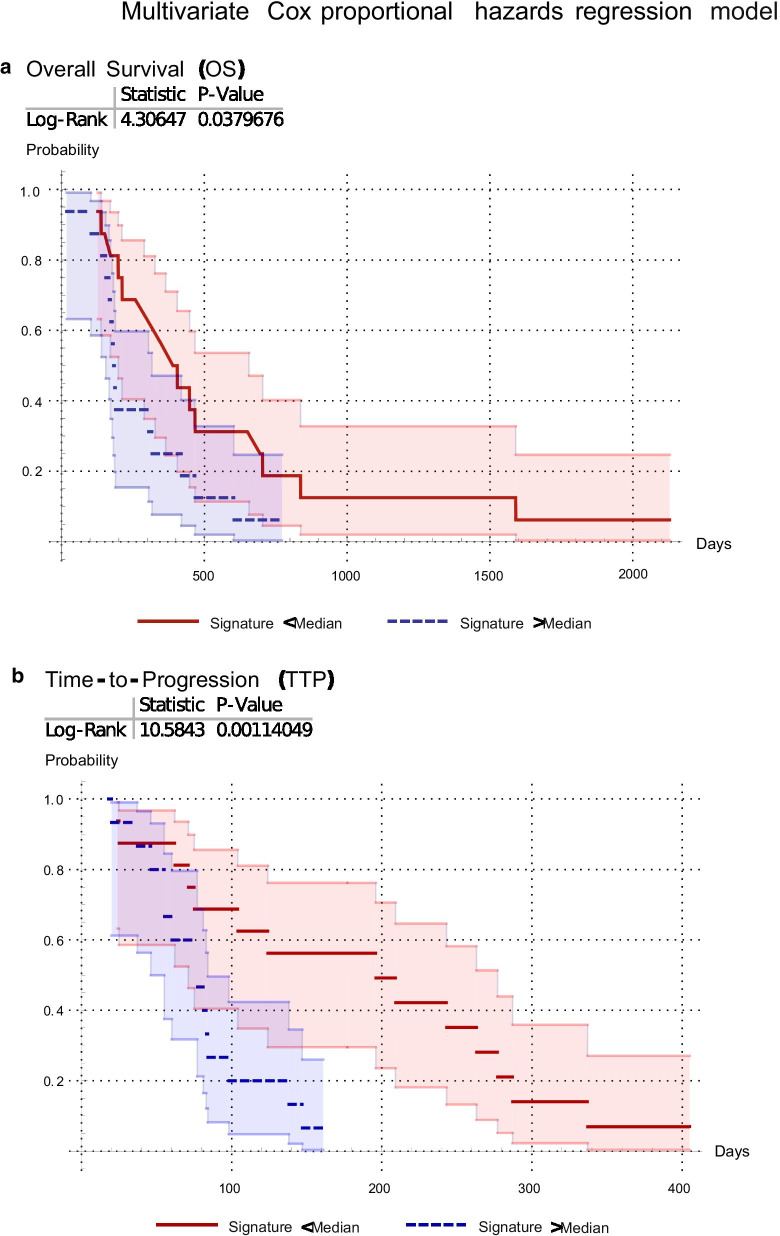
Kaplan–Meier curves and log-rank test for the radiomics-signatures predicting overall survival (OS-radiomics-signature) (**a**) and time-to-progression (TTP-radiomics-signature) (**b**)

The *p* value for all three overall tests (Likelihood-Ratio, Wald, and Score) was significant (*p* < 0.05) for both radiomics-signature models. In Fig. 2 the Kaplan–Meier curves and log-rank test for the resulted radiomics-signature models are shown. For the TTP-radiomics-signature, results from the Kaplan–Meier curve and log-rank test (Fig. 2b) showed the same performance as when only considering the texture feature SZLGE.

**Fig. 3:**
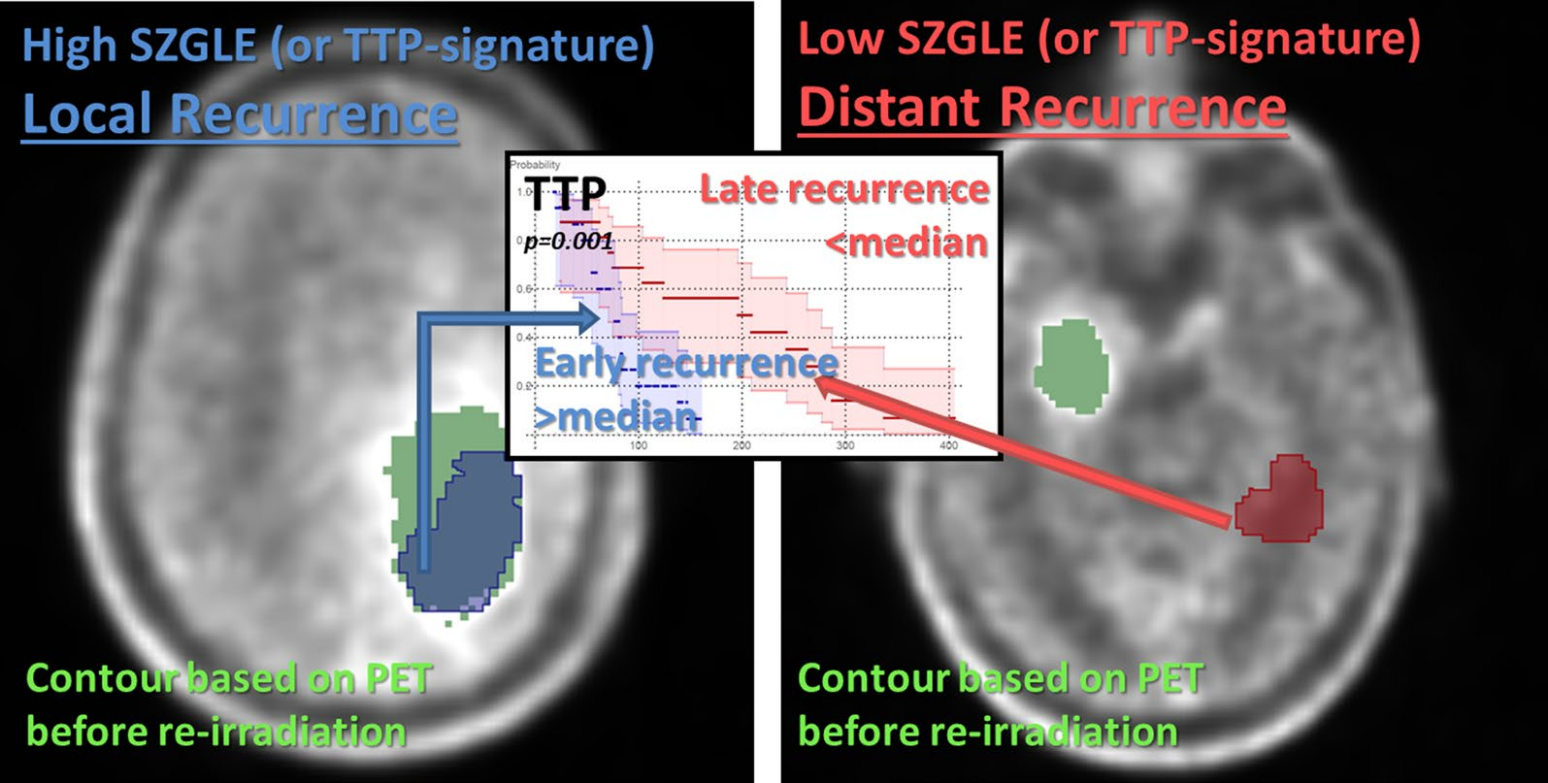
Scheme of the pattern observed for values of SZLGE and TTP-radiomics-signature in regards to the recurrence location (AUC_SZLGE_ = 0.63 and AUC_TTP-radiomics-signature_ = 0.66) and time-to-progression (Kaplan–Meier curves p_SZLGE_ = p_TTP-radiomics-signature_ = 0.001): high SZGLE for early in field recurrence after re-RT (blue) and low SZGLE for late distant recurrence after re-RT (red)

For TTP-radiomics-signature and SZLGE we additionally evaluated their power as predictors of RL. Results showed moderate RL predictions with an area-under-the-curve (AUC) and sensitivity of 0.66 and 0.78 for the TTP-radiomics-signature and 0.63 and 0.79 for SZLGE, respectively. Therefore, for TTP-signature and for SZLGE, high values indicated not only earlier progression (TTP), but also a RL within the initial FET-PET active volume (Fig. 3).

## Discussion

To our knowledge, this is the first evaluation of the prognostic role of FET-RF in patients with rGBM scheduled for re-irradiation. The feasibility of developing a prognostic model based on FET-RF could be demonstrated, with the texture feature Small-Zone-Low-Grey-Emphasis (SZLGE) showing the best performance for the prediction of time-to-progression (TTP) and in field versus distant RL. Considering that re-irradiation can be associated with side effects like radiation necrosis, brain edema, increased corticosteroid use or need for bevacizumab treatment, these results could have a significant clinical importance in the selection of suitable patients. These results are to be further validated in larger and multi-institutional patient cohorts (e.g. patients in the multicenter GLIAA trial [21]) and could therefore be considered for the development of future prognostic scores.

In the past decade, the number of radiomics publications has dramatically increased. However, few articles have properly addressed the unique challenges that a model development based on PET-radiomics may pose, as a result of the dependence of the RF variability on multiple factors. In the following, we detailed the main considerations applied in the methodology of this study in order to minimize redundancy and to maximize robustness of RF, as recommended in previous PET-radiomics guideline publications [34–37]. Firstly, the definition of RF was performed according to the Image Biomarker Standardization Initiative [23] and recommendations for resampling and 3D definition [26, 27] were followed in order to minimize the impact on RF variability caused by the definition of texture feature matrices [29]. Additionally, our in-house code was validated with the open source software MITK [24] and an open source MATLAB code [25]. It permits to easily reproduce the computation of the RF involved in our study. Secondly, the impact of different reconstruction parameters and scanner design on PET RF variability [38] was assessed with experimental phantom evaluation. The intrinsic dependence of some RF on the statistics implied by the number of voxels within the volume [36] was also evaluated with experimental phantoms. Consequently, only the RF robust with respect to the different PET/CT scanners and independent of the number of voxels within the contour were involved in the development of the prognosis model and in the evaluation of the tumor characterization, respectively. Thirdly, in order to minimize the well-known inter- and intra-user variability observed by manual contouring and therefore its impact on RF variability [26, 34], a semi-automatic segmentation algorithm was employed. In addition, the added value of the RF (not redundancy) with respect to the parameters conventionally used for tumor characterization (volume and SUV_max_) was assessed. Results showed that texture features provided complementary information with respect to these conventional parameters and therefore their evaluation in the design of prognostic models could be supported. Finally, the generalizability of the results has been evaluated by internal validation, using imbalance-adjusted bootstrap as a resampling strategy in model development [25] and a strong criterion for multiple test correction (Bonferroni) was applied in order to reject false positives.

Conventionally, radiomics for brain tumors has primarily focused on MRI. However, recent publications have also evaluated the use of FET-PET radiomics for differentiation of grade III and IV primary tumors [18], the diagnosis of pseudoprogression in high-grade glioma [19, 39] and the correlation with isocitrate dehydrogenase genotype [20]. In agreement with previous publications, results of our investigation confirmed that FET-PET can have a prognostic value in the field of neuro-oncology [6–14, 40]. We presented the first evaluation of FET-PET radiomics for the prognosis assessment of rGBM patients scheduled for re-irradiation. Although the size of our cohort was limited by its prospective character, it was comparable or larger than the cohorts evaluated in previous publications [36, 39].

The texture feature that showed the most statistically significant prognostic value was SZLGE derived from the high-risk volume V_PETmax_. More precisely, high values of SZLGE and of the TTP-radiomics-signature in the FET-PET tumor volume before re-irradiation were correlated with earlier recurrence and a localization of the recurrence within the gross-tumor-volume defined on PET. These patients would therefore probably benefit less from re-irradiation and could be considered for alternative therapies (surgery, chemotherapy) or irradiation dose escalation. Low values of SZLGE and of the TTP-radiomics-signature correlated with longer TTP and distant recurrences, which is consistent with previous publications showing longer progression-free survival in patients with non-local recurrences [41]. Furthermore, we also identified an OS-radiomics-signature with statistical significance (*p* = 0.038, Fig. 2).

From the two FET-PET quantification parameters with prognostic value (SZGLE and the TTP-radiomics-signature), SZGLE should be preferred because of its easier computation. In addition, SZGLE has been previously reported to be robust with respect to analogical/digital PET/CT systems, CT artefacts, segmentation methods and lesion motion [26, 42]. Consequently, the reproducibility of SZLGE for the PET/CT imaging protocols conveyed by different institutions could be expected. SZLGE quantifies the amount and the signal intensity of small zones of low uptake. SZLGE prediction of TTP and RL and its statistically significant discrimination between tumor and background suggested that small low FET-uptake regions may also play an important role in the characterization of aggressive tumors. In addition, SZLGE is derived from the gray-level size zone matrix (GLSZM [30, 32]), which has been previously reported to provide quantitative parameters for non-invasive prediction of the IDH genotype in gliomas [20].

The main limitation of our study was that our results could not be validated on a second patient cohort with similar characteristics. We attempted an internal validation by retrospective selection of rGBM patients treated at our institution. We were able to collect 22 patients who underwent the same FET-PET acquisition protocol, but clinical characteristics differed significantly from the original cohort: IDH status, use of surgery, delivered dose, follow-up time, percentage of patients with recurrence, etc. Consequently, this cohort was considered not appropriate for the validation of our findings and future validation in a larger prospective cohort with similar clinical characteristics is still required. However, it is worth mentioning that for the patients of the retrospective cohort with recurrence (45%), values for SZLGE and the TTP-radiomics-signature confirmed the pattern observed in the initial cohort (Fig. 3) and significance was achieved for the prediction of RL. Specifically, patients with distant recurrence presented low SZLGE (average 0.019 < median) and late progression (average 155 days, with 180 days for the initial cohort), while patients with local recurrence showed high SZLGE (average 0.035 > median) and early progression (average 94 days, with 77 days for the initial cohort). Furthermore, the TTP-radiomics-signature (and SZGLE) predicted RL with AUC = 0.68 (0.58) and sensitivity = 0.79 (0.84).

Future work will involve all the patients prospectively enrolled in the multicenter GLIAA trial [21] and will therefore allow an additional external validation of our preliminary results. After confirmation, SZLGE could therefore be suggested for the improvement of individualized patient and treatment selection in rGBM.

## Conclusions

We presented the first evaluation of the role of FET-PET RF in the prognosis of patients with rGBM scheduled for re-irradiation. Our findings have shown that the radiomic feature SZLGE and two TTP- and OS-radiomics-signatures could significantly distinguish re-irradiation responders from non-responders. Further analysis in a larger prospective validation cohort is warranted and planned.

## Supplementary information

**Additional file 1**. Table of radiomics features.

**Additional file 2**. Results of radiomics features analysis. Black box means positive result for the analysis described on the first row and represents the property of interest; like for example, RF robust to the different PET/CT systems (second column).

## Data Availability

The datasets during and/or analysed during the current study available from the corresponding author on reasonable request.

## Abbreviations

FET: O-(2-[18F]fluoroethyl)-L-tyrosine
PET: Positron emission tomography
rGBM: Recurrent glioblastoma
RF: Radiomics features
SZLGE: Small-Zone-Low-Gray-Level-Emphasis
TTP: Time-to-progression
LR: Local Recurrence
DM: Distant Metastasis
CT: Computed tomography
SUV: Standardized uptake value
SD: Standard deviation
RT: Radiation therapy
TF64: GEMINI TF TOF 64
BB: GEMINI TF 16 Big Bore
EANM: European Association of Nuclear Medicine
EARL: European Association Research Ltd
BLOB-OS-TF: LOR-based ordered-subset iterative time-of-flight algorithm using spherical coordinates
HR: Hazard ratios
re-RT: Re-irradiation
MRI: Magnetic resonance imaging
Gd-T1MR: Gadolinium contrast enhanced T1-weighted images
MITK: Medical Imaging Interaction Toolkit
WF: Wavelet band-pass filtering
Q: Quantization algorithm
NP: NEMA NU2 Phantom
AUC: Area-under-the-curve
