# Supplementary material for: FET-PET radiomics in recurrent glioblastoma: prognostic value for outcome after re-irradiation?
Source: Radiat Oncol. 2021 Mar 3;16:46. doi: 10.1186/s13014-020-01744-8 (PMC7931514; doi:10.1186/s13014-020-01744-8)
Supplement: Supplementary file 1 — Additional file 1. Table of radiomics features. [file 13014_2020_1744_MOESM1_ESM.docx]

| SUV-Histogram | Maximum SUV (SUV_max_), Peak SUV (SUV_peak_), Minimum SUV (SUV_max_), |
| --- | --- |
|  | Mean SUV (SUV_mean_), Volume (V), Total Lesion FET (TL-FET),  Area under the curve of the cumulative SUV-volume histogram (aucCSH),  Skewedness (S), Coefficient of variance (CoV), Kurtosis (K),  Integral Uniformity (IU), Differential Uniformity (DU),  Energy(E_H_) and Entropy (Ent_H_), |
| Geometry | Solidity (S), Eccentricity (Ecc ), Long Diameter (LD), Percent Inactive (PI) |
| GLCM | Local Homogeneity (LH), Correlation (C_CM_ ), Contrast (Con_CM_), |
|  | Energy (E_CM_), Entropy (Ent_CM_), Variance (Var_CM_ ),  Autocorrelation (Acor ) and Dissimilarity (D) |
| GLRLM | Short Run Emphasis (SRE), Long Run Emphasis (LRE), |
|  | Low Gray-Level Run Emphasis (LGRE), High Gray-Level Run Emphasis (HGRE) |
|  | Short Run Low Gray-Level Emphasis (SRLGE), Short Run High Gray-Level  Emphasis (SRHGE), Long Run Low Gray-Level Emphasis (LRLGE),  Long Run High Gray-Level Emphasis (LRHGE),  Gray-Level Non-uniformity (GLN), Run Percentage (RP)  Length Non-uniformity (RLN) |
| GLSZM | Small Zone Emphasis (SZE), Large Zone Emphasis (LZE), |
|  | Low Gray-Level Zone Emphasis (LGZE), High Gray-Level Zone Emphasis (HGZE),  Small Zone Low Gray-Level Emphasis (SZLGE), Small Zone High  Gray-Level Emphasis (SZHGE), Large Zone Low Gray-Level Emphasis(LZLGE),  Large Zone High Gray-Level Emphasis (LZHGE), |
|  | Zone-Size Non-uniformity (ZSN), Zone Percentage (ZP),  Gray-Level Variance (GLV) and Zone-Size Variance (ZSV) |
| NGTDM | Coarseness (Coar), Contrast (Con_NM_), |
|  | Complexity (Comp) and Texture Strength (TS) |
|  |  |

**ESM Table 1. Image Features**

Abbreviations: SUV: standardized uptake values, GLCM: gray-level co-occurrence matrix, GLRLM: gray-level run length matrix, GLSZM: gray-level size zone matrix, NGTDM: neighborhood gray tone difference matrix.
