# Supplementary material for: FET-PET radiomics in recurrent glioblastoma: prognostic value for outcome after re-irradiation?
Source: Radiat Oncol. 2021 Mar 3;16:46. doi: 10.1186/s13014-020-01744-8 (PMC7931514; doi:10.1186/s13014-020-01744-8)
Supplement: Supplementary file 2 — Additional file 2. Results of radiomics features analysis. Black box means positive result for the analysis described on the first row and represents the property of interest; like for example, RF robust to the different PET/CT systems (second column). [file 13014_2020_1744_MOESM2_ESM.docx]

**ESM Table 2.** Results of RF Analysis. Black box means positive result for the analysis described on the first row and represents the property of interest; like for example, RF robust to the different PET/CT systems (second column).

| **RF** | **Robust (BB vs TF64)** | **Non-dependent**  **on size**  **(voxels)** | **Tumor Discrimination**  **(V_PETmax_)** | **Tumor Discrimination**  **(V_PET3mm_)** | **RF(V_PETmax_)**  **non-correlated with**  **volume and SUV_max_** | **RF(V_PET3mm_)**  **non-correlated with**  **volume and SUV_max_** |
| --- | --- | --- | --- | --- | --- | --- |
| % | 61 | 53 | 75 | 21 | 48 | 45 |
| V | 1 | 0 | NA | NA | 0 | 0 |
| SUV_max_ | 1 | 1 | 1 | 0 | 0 | 0 |
| TL-FET | 1 | 0 | NA | NA | 0 | 0 |
| SUV_peak_ | 1 | 1 | 1 | 0 | 0 | 0 |
| SUV_mean_ | 1 | 1 | 1 | 1 | 1 | 1 |
| aucCSH | 1 | 1 | 1 | 1 | 1 | 1 |
| SUVmin | 1 | 1 | 1 | 1 | 1 | 1 |
| CoV | 0 | 1 | 0 | 1 | 1 | 1 |
| Skewness | 1 | 1 | 1 | 1 | 1 | 1 |
| Kurtosis | 1 | 1 | 0 | 0 | 1 | 1 |
| EntropyLog2 | 1 | 1 | 0 | 0 | 1 | 1 |
| Energy (E_H_) | 1 | 1 | 1 | 1 | 1 | 1 |
| IU | 1 | 0 | NA | NA | 1 | 0 |
| DU | 1 | 0 | NA | NA | 0 | 0 |
| Solidity | 1 | 0 | NA | NA | 1 | 1 |
| Eccentricity | 1 | 1 | 1 | 0 | 1 | 1 |
| LD | 1 | 0 | NA | NA | 0 | 0 |
| PI | 1 | 0 | NA | NA | 0 | 0 |
| Energy_CM_ | 0 | 1 | 0 | 0 | 0 | 0 |
| Contrast_CM_ | 0 | 1 | 1 | 0 | 1 | 1 |
| Entorpy_CM_ | 0 | 1 | 0 | 0 | 0 | 0 |
| LH | 0 | 1 | 1 | 0 | 1 | 1 |
| Correlation_CM_ | 1 | 1 | 1 | 1 | 0 | 0 |
| Variance_CM_ | 1 | 1 | 1 | 1 | 1 | 1 |
| D | 0 | 1 | 1 | 0 | 1 | 1 |
| Acor | 0 | 0 | NA | NA | 0 | 0 |
| SZE | 0 | 1 | 1 | 0 | 1 | 1 |
| LZE | 0 | 0 | NA | NA | 1 | 1 |
| GLN | 0 | 1 | 0 | 0 | 0 | 0 |
| ZSN | 0 | 1 | 1 | 0 | 1 | 1 |
| ZP | 0 | 1 | 1 | 0 | 1 | 1 |
| LGZE | 1 | 0 | NA | NA | 1 | 0 |
| HGZE | 0 | 0 | NA | NA | 0 | 0 |
| **SZLGE** | 1 | 1 | 1 | 0 | 1 | 0 |
| SZHGE | 0 | 1 | 1 | 1 | 0 | 0 |
| LZLGE | 1 | 1 | 0 | 1 | 1 | 0 |
| LZHGE | 1 | 0 | NA | NA | 0 | 0 |
| GLV | 1 | 0 | NA | NA | 0 | 1 |
| ZSV | 0 | 0 | NA | NA | 0 | 0 |
| SRE | 0 | 1 | 1 | 0 | 1 | 1 |
| LRE | 0 | 1 | 1 | 0 | 1 | 1 |
| GLN2 | 0 | 1 | 0 | 0 | 0 | 0 |
| RLN | 0 | 1 | 1 | 0 | 1 | 1 |
| RP | 0 | 1 | 1 | 0 | 1 | 1 |
| LGRE | 1 | 0 | NA | NA | 1 | 0 |
| HGRE | 0 | 0 | NA | NA | 0 | 0 |
| SRLGE | 1 | 0 | NA | NA | 1 | 0 |
| SRHGE | 0 | 0 | NA | NA | 0 | 0 |
| LRLGE | 1 | 1 | 1 | 0 | 1 | 0 |
| LRHGE | 0 | 0 | NA | NA | 0 | 0 |
| GLV2 | 1 | 1 | 0 | 1 | 0 | 1 |
| RLV | 1 | 0 | NA | NA | 0 | 0 |
| Coarseness | 1 | 0 | NA | NA | 0 | 0 |
| Contrast_NM_ | 0 | 1 | 1 | 0 | 1 | 1 |
| Busyness | 1 | 0 | NA | NA | 1 | 1 |
| Complexity | 0 | 0 | NA | NA | 0 | 0 |
| TS | 0 | 0 | NA | NA | 1 | 1 |
| WF_E_CM_ | 1 | 1 | 0 | 0 | 0 | 0 |
| WF_Con_CM_ | 0 | 1 | 1 | 0 | 0 | 1 |
| WF_Ent_CM_ | 1 | 1 | 0 | 0 | 0 | 0 |
| WF_LH | 0 | 1 | 1 | 0 | 1 | 1 |
| WF_C_CM_ | 1 | 1 | 0 | 0 | 0 | 0 |
| WF_Var_CM_ | 1 | 1 | 1 | 0 | 1 | 1 |
| WF_D | 0 | 1 | 1 | 0 | 0 | 1 |
| WF_Acor | 0 | 0 | NA | NA | 0 | 0 |
| WF_SZE | 0 | 1 | 1 | 0 | 1 | 1 |
| WF_LZE | 0 | 0 | NA | NA | 1 | 1 |
| WF_GLN | 1 | 1 | 0 | 0 | 0 | 0 |
| WF_ZSN | 0 | 1 | 1 | 0 | 1 | 1 |
| WF_ZP | 0 | 1 | 1 | 0 | 1 | 1 |
| WF_LGZE | 1 | 0 | NA | NA | 0 | 0 |
| WF_HGZE | 0 | 0 | NA | NA | 0 | 0 |
| WF_SZLGE | 1 | 1 | 0 | 0 | 0 | 0 |
| WF_SZHGE | 0 | 1 | 1 | 1 | 0 | 0 |
| WF_LZLGE | 1 | 1 | 0 | 0 | 0 | 0 |
| WF_LZHGE | 1 | 0 | NA | NA | 1 | 0 |
| WF_GLV | 1 | 0 | NA | NA | 0 | 1 |
| WF_ZSV | 1 | 0 | NA | NA | 0 | 0 |
| WF_SRE | 0 | 1 | 1 | 0 | 1 | 1 |
| WF_LRE | 0 | 1 | 1 | 0 | 1 | 1 |
| WF_GLN2 | 1 | 1 | 1 | 0 | 0 | 0 |
| WF_RLN | 0 | 1 | 1 | 0 | 1 | 1 |
| WF_RP | 0 | 1 | 1 | 0 | 1 | 1 |
| WF_LGRE | 1 | 0 | NA | NA | 0 | 0 |
| WF_HGRE | 0 | 0 | NA | NA | 0 | 0 |
| WF_SRLGE | 1 | 0 | NA | NA | 0 | 0 |
| WF_SRHGE | 0 | 0 | NA | NA | 0 | 0 |
| WF_LRLGE | 1 | 1 | 0 | 0 | 0 | 0 |
| WF_LRHGE | 0 | 0 | NA | NA | 0 | 0 |
| WF_GLV2 | 1 | 1 | 0 | 0 | 0 | 1 |
| WF_RLV | 1 | 0 | NA | NA | 0 | 0 |
| WF_Coar | 1 | 0 | NA | NA | 0 | 0 |
| WF_Con_NM_ | 0 | 1 | 1 | 0 | 1 | 1 |
| WF_B | 1 | 0 | NA | NA | 1 | 1 |
| WF_Comp | 0 | 1 | 1 | 0 | 0 | 0 |
| WF_TS | 1 | 0 | NA | NA | 1 | 1 |
| QEnergy_CM_ | 1 | 0 | NA | NA | 0 | 1 |
| QContrast_CM_ | 1 | 1 | 1 | 1 | 1 | 1 |
| QEntorpy_CM_ | 1 | 0 | NA | NA | 0 | 1 |
| QLH | 0 | 1 | 1 | 0 | 1 | 1 |
| QC_CM_ | 1 | 1 | 0 | 0 | 0 | 0 |
| QVariance_CM_ | 1 | 1 | 1 | 1 | 1 | 1 |
| QD | 1 | 1 | 1 | 0 | 1 | 1 |
| QAcor | 1 | 0 | NA | NA | 1 | 1 |
| QSZE | 1 | 1 | 1 | 0 | 1 | 0 |
| QLZE | 0 | 1 | 1 | 0 | 0 | 0 |
| QGLN | 1 | 0 | NA | NA | 0 | 1 |
| QZSN | 1 | 1 | 1 | 0 | 1 | 0 |
| QZP | 1 | 1 | 1 | 0 | 0 | 0 |
| QLGZE | 1 | 0 | NA | NA | 1 | 0 |
| QHGZE | 1 | 0 | NA | NA | 1 | 1 |
| QSZLGE | 1 | 0 | NA | NA | 0 | 0 |
| QSZHGE | 1 | 0 | NA | NA | 1 | 1 |
| QLZLGE | 1 | 1 | 1 | 0 | 1 | 1 |
| QLZHGE | 1 | 0 | NA | NA | 0 | 0 |
| QGLV | 0 | 0 | NA | NA | 0 | 0 |
| QZSV | 1 | 0 | NA | NA | 0 | 0 |
| QSRE | 0 | 1 | 1 | 0 | 0 | 0 |
| QLRE | 0 | 1 | 1 | 0 | 0 | 0 |
| QGLN2 | 1 | 0 | NA | NA | 0 | 1 |
| QRLN | 0 | 1 | 1 | 0 | 0 | 0 |
| QRP | 0 | 1 | 1 | 0 | 0 | 0 |
| QLGRE | 1 | 0 | NA | NA | 0 | 0 |
| QHGRE | 1 | 0 | NA | NA | 1 | 1 |
| QSRLGE | 1 | 0 | NA | NA | 0 | 0 |
| QSRHGE | 1 | 0 | NA | NA | 1 | 1 |
| QLRLGE | 1 | 1 | 1 | 0 | 0 | 0 |
| QLRHGE | 1 | 0 | NA | NA | 0 | 1 |
| QGLV2 | 1 | 0 | NA | NA | 1 | 0 |
| QRLV | 0 | 0 | NA | NA | 0 | 0 |
| QCoar | 1 | 0 | NA | NA | 0 | 0 |
| QCont_NM_ | 1 | 1 | 1 | 1 | 1 | 0 |
| QB | 1 | 0 | NA | NA | 1 | 0 |
| QComp | 1 | 0 | NA | NA | 1 | 1 |
| QTS | 1 | 0 | NA | NA | 1 | 0 |
